# Experimental and Numerical Analysis of Chlorinated Polyethylene Honeycomb Mechanical Performance as Opposed to an Aluminum Alloy Design

**DOI:** 10.3390/ma15228034

**Published:** 2022-11-14

**Authors:** Florin Baciu, Anton Hadăr, Andrei-Daniel Voicu, Daniel Vlăsceanu, Daniela-Ioana Tudose

**Affiliations:** 1 Department of Strength of Materials, Faculty of Industrial Engineering and Robotics, University Politehnica of Bucharest, 313 Splaiul Independenței, Sector 6, 060042 Bucharest, Romania; 2 Academy of Romanian Scientists, 3 Ilfov Street, Sector 5, 050045 Bucharest, Romania; 3 Technical Sciences Academy of Romania, 26 Dacia Boulevard, Sector 1, 030167 Bucharest, Romania

**Keywords:** helicopter blade, tensile test, orthotropic study, bare compression test, fused deposition modeling, sandwich structure, Digital Image Correlation, honeycomb, 5052 aluminum alloy, polyethylene

## Abstract

Manufacturing aircraft components through 3D printing has become a widespread concept with proven applicability for serial production of certain structural parts. The main objective of the research study is to determine whether a chlorinated polyethylene material reinforced with milled carbon fibers has the potential of replacing the current 5052 NIDA aluminum alloy core of the IAR330 helicopter tail rotor blade, under the form of a honeycomb structure with hexagonal cells. Achieving this purpose implied determining the tensile and compression mechanical properties of the material realized by fused deposition modeling. The tensile tests have been conducted on specimens manufactured on three printing directions, so that the orthotropic nature of the material may be taken into account. The bare compression tests were realized on specimens manufactured from both materials, with similar honeycomb characteristics. All the mechanical tests have been performed on the Instron 8872 servo hydraulic testing system and the results have been evaluated with the Dantec Q400 Digital Image Correlation system. The experimental tests have been reproduced as finite element analyses which have been validated by results comparison, in order to determine if the compression model is viable for more complex numerical analysis.

## 1. Introduction

Composite materials are of fundamental importance in the aviation industry, being a key element in the development of modern aircraft. They greatly enhance the overall performance of aircrafts, mainly by weight reduction, but also by improved mechanical properties. 

Although composite materials have been embedded in the structural design aircraft for several decades, there is still in use a large number of aircrafts made predominantly from metal alloys, especially aluminum. Such is the case of the IAR330 multirole helicopter, still in active use by the Romanian Air Force and with a foreseen lifespan of approximately ten more years to come. The tail rotor blades of the above-mentioned helicopter are made from a combination of aluminum alloys and would benefit from a conversion towards composite materials. The aim of the research is to evaluate if a specific chlorinated polyethylene material is suitable for replacing the 5052 aluminum alloys used to manufacture the honeycomb core of the tail rotor blade and to underline the main advantages and disadvantages. 

Composite aviation structures are not a novelty in the aircraft fabrication. Over the years, many metal components have been replaced with composite materials which outperform them. The percentage of composites used is aircraft has evolved from approximately 6% in 1985 to over 50% in the last decade [1]. The necessity for weight reduction is higher in helicopters than in airplanes, due to the fact they the engines must support the entire mass of the helicopter. Thus, newer generation helicopters, such as the Eurocopter Tiger or the NH90, manufactured by NHIndustries with the headquarters in Aix-en-Provence, France, have a composite material constitution of over 80%, with major proportions in the aircraft fuselage, in the main rotor hub and blades, but also in the interior furnishing components [2].

Maintenance procedures are very important in aircraft operation, due to the expensive nature of the inspection/repair operations and also, due to the fact that aircrafts undergo a strict maintenance routine as to respect flight schedules. Composite materials have the potential of relieving this burden, by transitioning from the hourly lifespan of life-limited parts, to “on-condition” maintenance. Traditional aircraft materials require frequent maintenance inspections in order to ensure a high degree of aircraft safety. This is the reason why certain manufacturers, such as Airbus, have introduced composite components, which generally have higher acquisition costs, but require less planned maintenance [3,4].

The quest for better performances determined manufacturers and operators alike to find cost effective solutions for replacing traditional metal structures with composite ones [5,6], which present a higher lifespan, improved resistance to mechanical stress and also a significant weight loss, which is crucial in the aerospace industry. One of the main objectives of the study is to highlight the importance of making the transition towards composite aviation structures, by highlighting the benefits in terms of resistance and mass reduction.

Components realized by fused deposition modeling (FDM) are becoming a tangible reality in recent aircraft models. Although it might take time for this manufacturing technique to standardize and to demonstrate its airworthiness, there will certainly come a moment in the near future when spontaneous demands for aircraft parts will be satisfied through this manufacturing method. The increasing attention received by additive manufacturing techniques can be clearly seen in the recent years’ trends [7], as the number of related scientific publications has steadily increased, FDM being at the forefront of research topics.

Major industrial manufacturers, such as General Electric, have dedicated entire departments to produce aircraft components through additive manufacturing, such as the turbine ring for the future FATE engine or the fuel nozzles for the LEAP-1A engines [8]. A hybrid structure approach can be foreseen as 3D printed parts will work together with traditionally manufactured components to enhance performances and to reduce aircraft out-of-service time. Recently, the world’s first jet-powered UAV made from 80% 3D printed components was developed by Aurora Flight Sciences and Stratasys from a lightweight material called ULTEM™ 9085 [9]. Other recent aircraft implementations include wing prototypes [10], fuselage components of different shapes and sizes [11] and other different functional parts [12]. An important milestone has been surpassed in terms of certified production by the company Materialise, which has multiple facilities with commercial grade FDM machines, being certified as a Production Organization in accordance with EASA Part 21G [13].

**Figure 1:**
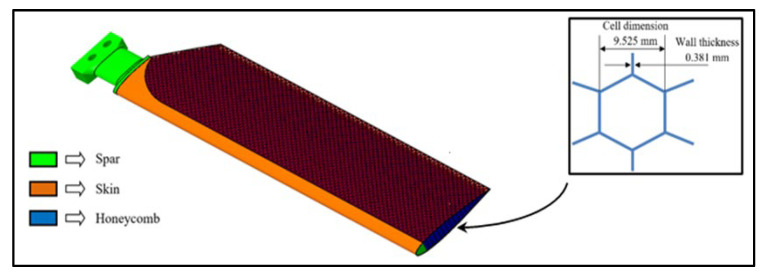
Main components of the composite tail rotor blade model.

The composite model of the blade has been designed to encompass a carbon fiber roving spar, a carbon fabric reinforced composite for the skin and a lightweight 3D printed honeycomb core. The honeycomb interior, which is of interest for the current study, represents an array of hexagonal cells made from chlorinated polyethylene (CPE) with milled carbon fibers, manufactured through a fused deposition modeling (FDM) process. The commercial name of the material is CPE CF112 Carbon. Together with the upper and lower skin, they form a sandwich-structured composite which offers excellent strength to external loads. The main structural components of the IAR330 tail rotor blade are depicted in Figure 1, with a detail on the cell dimensions. The mechanical properties of the composite laminated material used for the skin of the blade were rigorously studied and presented in a previous article [14]. Also, other cellular materials have been studied in order to evaluate which might be the best option for the future version of the composite blade [15,16].

The Dantec Q400 Digital Image Correlation (DIC) system manufactured by Dantec Dynamics A/S located in Skovlunde, Denmark, uses an optical methodology for measuring displacements and strains in a material under external load, by tracking the visual changes that occur in the successive images memorized by the Istra 4D software program, developed also by Dantec Dynamics A/S [17]. Effective for both 2D and 3D measurements, the DIC system has a higher degree of efficiency compared to both extensometers or strain gages, due to their capacity to offer both local and global data. Applicable for metal alloys [18], composite materials [19] and more recently on 3D printed materials [20], the methodology has demonstrated its practical capabilities.

**Figure 2:**
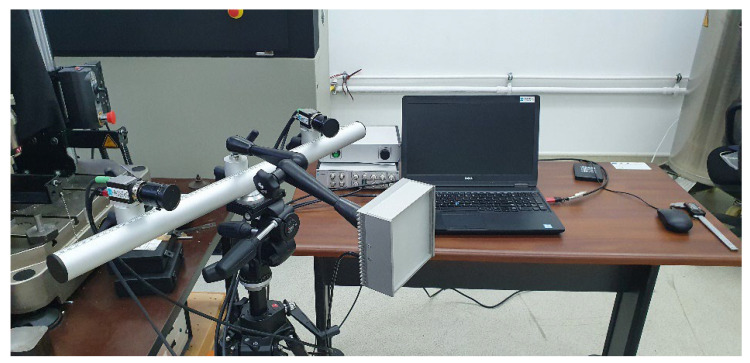
The Q400 Digital Image Correlation setup.

The Q400 system gives the user the capability of a 3D full field in order to measure deformation, strain, shape and vibration on almost any material and any shape, by comparing succeeding images of a test specimen’s surface, recorded by the two cameras. The device utilizes a special monochromatic illumination in order to improve accuracy and repeatability of the measurements. Finite element analyses performed in powerful software programs such as Ansys Workbench, developed by ANSYS Inc. in Canonsburg, PA, USA, gives users the capability of assessing the performance of complex structures. The two cameras of the Digital Image Correlation setup as well as the monochromatic illumination lamp are orientated towards the Instron servo hydraulic testing system, as can be seen in Figure 2.

In order to perform this method, the test specimens must be marked with a random pattern of contrasting colors. The exposed surfaces of the specimens where first spray painted white, after which a random pattern of black dots was applied.

The current article aims at obtaining both a tensile and a compressive characterization of the material utilized for the honeycomb core, realized by fused deposition modeling. The experimental results shall constitute a basis for validation the numerical simulations, so that the results can be utilized, in a larger extent, to evaluate the mechanical behavior of the composite tail rotor blade model subjected to aerodynamic loads which have been previously determined and validated experimentally [21].

Few studies are available on the topic of evaluating the compressive mechanical characteristics of a 3D printed honeycomb core with hexagonal cells, a fact which makes this subject all the more relevant. Honeycomb cores are a prefer solution for certain aviation components due to the significant mass reduction they induce [22]. Placed in both metal and composite structures under the form of sandwich structures, they provide the necessary strength and stiffness required in lightweight aircraft components. The mechanical characteristics of the honeycomb core generally depends on three factors: the physical properties of the material, the relative density and the geometrical shape of the cells. Frequently employed shapes include hexagonal cells, triangular cells, square cells and other similar variations. Studies which present certain similarities refer to tensile and compressive properties determination of different 3D printed honeycomb structures [23,24,25], the additive manufacturing of aircraft spare components [26] and the also mechanical evaluation of different sandwich structures with honeycomb cores, realized by additive manufacturing [27,28]. Also, due to the widespread use of the 5052-aluminum alloy as an aviation structural material, there are many references regarding its properties [29,30].

Polyethylene is an easily procurable commercial material which has been analyzed from different perspectives to assess its resistance enhancement capabilities [31,32]. It is one of the most popular thermoplastic materials, being available both in film and fabric form, for a multitude of applications such as: plastic containers, bags, etc. The chlorination process of the polyethylene enhances greatly its impact and weather resistance performances [33]. The strength of the material is further increased by the presence of milled carbon fibers. An alternative solution for enhancing the strength of the tail rotor blade would be to reinforce the aluminum alloys with nanoparticles, as certain studies might suggest [34], but the main objective of the study was to develop a new composite structure for the blade, that would imply more advantages.

## 2. Materials and Methods

### 2.1. Material Presentation

The material structure for the helicopter blade is conceived in terms of weight reduction and resistance to aerodynamic loads. The filament material used to print the honeycomb represents the lighter component and is designed to withstand long-term loads, presenting excellent wear and chemical resistance, in conjunction with great printability and dimensional stability.

The aluminum alloy utilized in manufacturing the core of the currently used IAR330 tail rotor blade is a NIDA-core structural honeycomb, produced by the company IAR in Brașov, Romania, and defined by the following characteristics:

- Honeycomb cell density—2.3 lbs/ft3, the equivalent of 36,842 kg/m^3^;
- Hexagonal cell with the following dimensions—3/8 inch, corresponding to 9.525 mm in metric units;
- Wall thickness—0.0015 inch, the equivalent of 0.381 mm.

The aluminum alloy 5052 is described as having a very high corrosion resistance, making it suitable for use in marine environments. The high resistance of the material is partially a result of the high magnesium concentration, making it one of the most resistant of its class. The material has a very good strength to weight ratio, being ideal for aerospace structures. A few of its other main advantages include: good performance at high temperatures, up to 180 °C, good fire resistance, high resistance to environmental factor, great malleability and manufacturability. The main mechanical characteristics of the two honeycomb materials, as presented by the manufacturer, are depicted in Table 1. A selection of the most important properties has been presented, according to the nature of each material type.

### 2.2. Tensile Testing—Experimental Set-Up and Methodology

Three printing directions were considered, with five specimens manufactured in each direction, in order to take into account, the implications of the orthotropic nature of additive manufactured materials. The dog-bone-shaped tensile test specimens have been realized in accordance with the ASTM 3039M-14 Standard test method for determining the tensile properties of composite materials [37]. The 3D model of the specimen was imported in the software used by the 3D printing machine.

Due to the fact that the CPE CF112 Carbon has milled carbon fibers, they have the potential of enhancing the mechanical resistance of the material in a certain direction, depending on their orientation. Over-hangings were utilized, when the situation demanded it, to support the 3D printed structures as to obtain the desired geometry. Out of the five specimens manufactured for each direction, a total of four have been evaluated by using Instron 8872 measuring system, coupled with the digital extensometer, whilst one has been assessed by means of Digital Image Correlation, in order to determine Poisson’s ratio.

**Figure 3:**
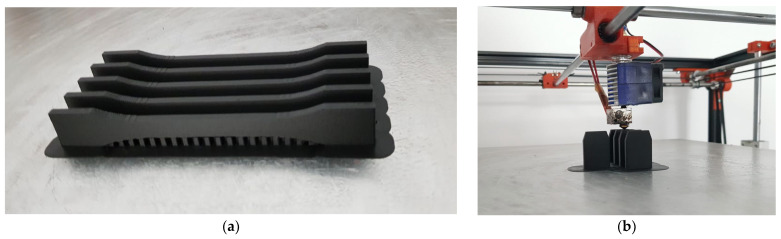
Different phases in the manufacturing process: (**a**) Tensile specimens printed on the lateral side, with over hangings; (**b**) Tensile test specimens printed along the vertical direction.

Figure 3 presents the fabrication of the tensile test specimens in several stages. Both phases reveal the first ultrathin laminar layer printed on the flat metal pad, which facilitates the detachment of the specimen when the model has been fully printed.

**Figure 4:**
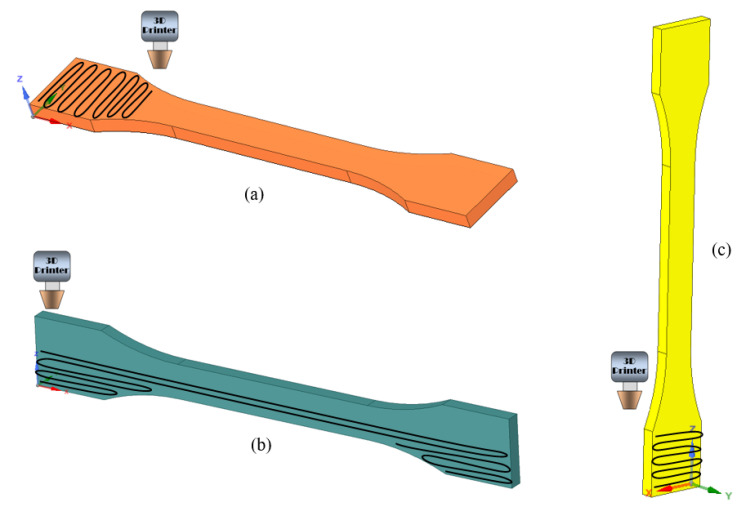
Tensile specimens printing directions: (**a**) XY-Specimen (printed flatwise); (**b**) XZ1-Specimen (printed sidewise); (**c**) XZ2-Specimen (printed lengthwise).

Considering XY geometric plane as coincident with the printing plane of the machine, the following three printing directions were defined in order to analyze the orthotropic nature of the fused deposition modeling manufacturing process, as presented in Figure 4.

**Figure 5:**
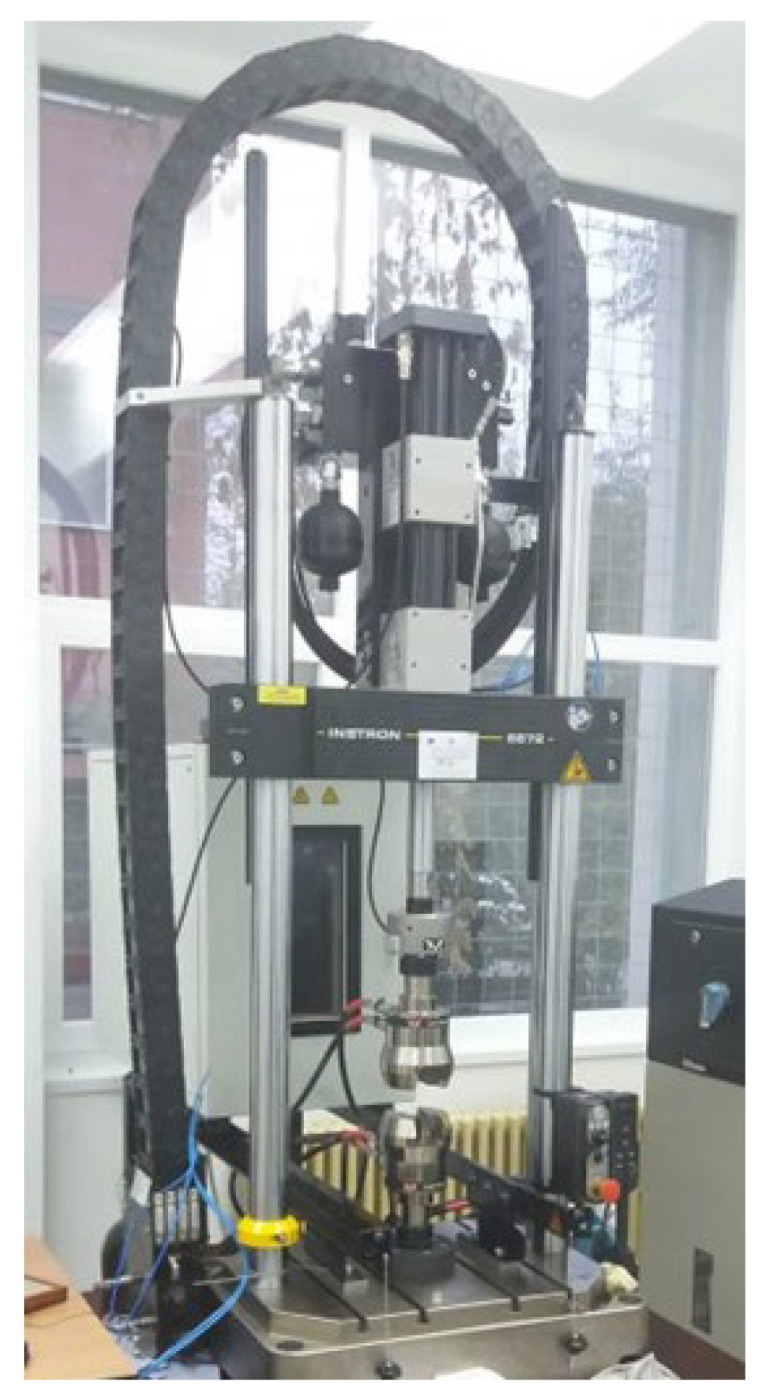
The Instron 8872 servo hydraulic testing system [38].

The tensile tests were realised using the Instron 8872 servo hydraulic testing system for axial static and dynamic tests presented in Figure 5, with the actuating cylinder and the 25 kN load cell being positioned in the upper side of the mobile section. The test conditions setup and the results interpretation were achieved with the Instron WaveMatrix testing software, developed by the company Instron in Norwood, MA, USA.

All the tensile test specimens were placed inside the hydraulic locking system of the machine and a jaw speed of 2 mm/min was applied, in order to obtain a constant strain rate in the gage section.

Environmental testing conditions consisted of the following parameters:

- Low exposure to UV radiation;
- Ambient temperature of 22 °C;
- Relative humidity of the air—under 60%.

The area of the central section of each tensile specimen was carefully measured at three sections in the gage segment and the average value inputted in the operating software. The specimens where then placed in the hydraulic grips of the testing machine aligned with the test direction. A precise digital extensometer with a work span of ±5 mm was attached to the test specimens in order to determine strain related characteristics. This instrument was removed before the specimen failure occurred, given that the fracture might also deteriorate the extensometer.

The tensile test specimens were 3D printed by using a Custom 3D Xcub printer, manufactured by Fibonacci Printing Solutions in Bucharest, Romania. The thermoplastic extrusion of the CPE Carbon fiber filament took place at to over 250 °C, in order to manufacture the desired specimens. The 3D printer used the following settings for manufacturing the tensile test specimens:

- printing resolution of 0.2 mm, to ensure the preservation of the initial geometrical characteristics, whilst reducing the manufacturing time to an acceptable value;
- 100% infill density, so that the samples are not hollow in any point;
- two hours printing duration, encompassing the time taken by the machine to convert the filament into the specific modelled structure;
- the rectangular pad of the printer was heated to a temperature of 30 °C to enhance specimen properties and to facilitate the manufacturing process;
- total material consumption recorded was 11 g of filament per test specimen.

**Figure 6:**
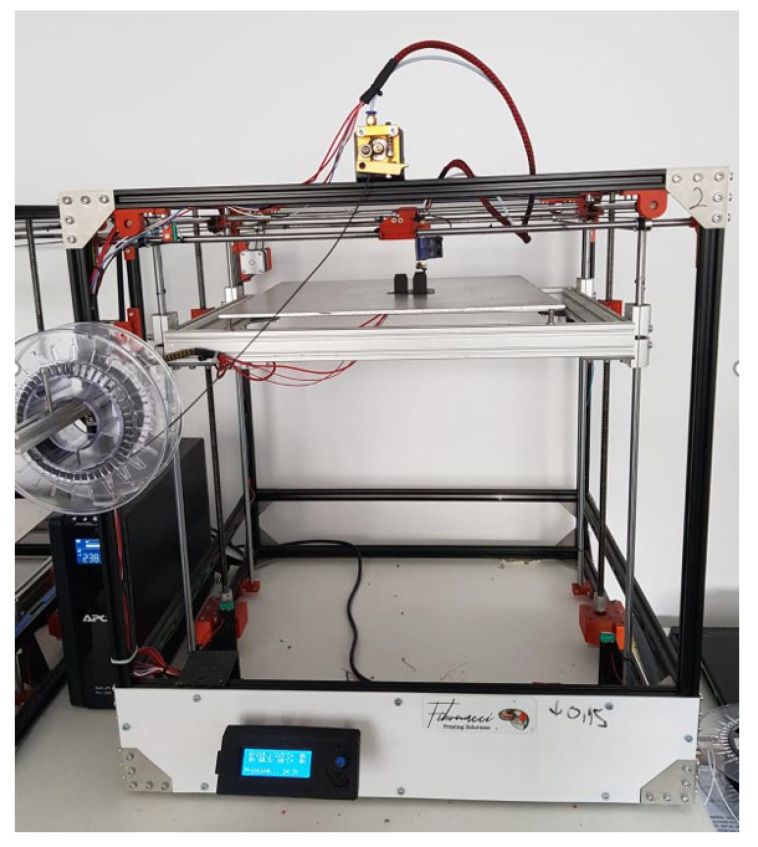
The Custom 3D Xcub printer employed for realizing the tensile test specimens.

A brief description of the manufacturing capabilities of the 3D printed displayed in Figure 6 are also presented in Table 2.

The tensile stress can be determined at each measured data point with the following formula:

(1) $$\sigma_{i}\;=\;\frac{P_{i}}{A}$$

where “*P_i_*” is the determined forced at each data point (measured in N), and “*A*” is the average cross-section area of the specimen (measured in mm^2^).

Based on the previous formula, the ultimate tensile strength can also be determined, if the maximum force before failure “*P_max_*” is know:

(2) $$\sigma_{tu}\;=\;\frac{P_{max}}{A}$$

In order to determine the tensile modulus, the strain of the material must be first determined. Thus, for each determined data point where the displacement has been determined by the extensometer ($\delta_{i}$) and knowing its gage length ($L_{g}$), the tensile strain can be determined using the expression:

(3) $$\varepsilon_{i}\;=\;\frac{\delta_{i}}{L_{g}}$$

Having determined the stress-strain parameters, the tensile modulus of elasticity can be determined as the slope of the stress-strain curve:

(4) $$E\;=\;\frac{\Delta \sigma }{\Delta \varepsilon }$$

The amount of transversal elongation ($\varepsilon_{transversal}$) divided by the amount of axial compression ($\varepsilon_{axial}$), was determined using the Q400 Digital Image Correlation system, with the following formula:

(5) $$\nu \;=\;-\frac{\varepsilon_{transversal}}{\varepsilon_{axial}}$$

### 2.3. Bare Compression Testing—Experimental Set-Up and Methodology

**Figure 7:**
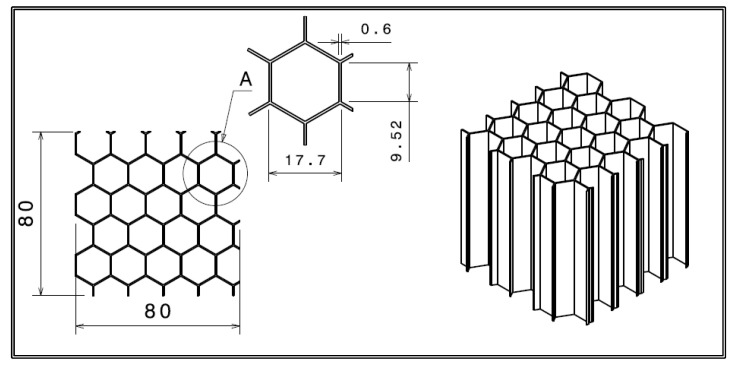
Dimensions of the compression cube specimen.

The experimental tests were realized in accordance with the ASTM C365-03 standard test method for determining flatwise compressive properties in sandwich cores. Due to the fact that the honeycomb specimens are not stabilized, the test is known as bare compression test [39]. The same ambient characteristics were recorded as for the tensile testing. Four CPE compression specimens were employed in this experimental test sequence, as well as eight 5052 aluminum alloy test specimens. The geometrical characteristics of the cubic shaped specimens manufactured from CPE are presented in Figure 7.

The ASTM standard implies that specimens with at least 5800 mm^2^ shall be tested, when material cells are larger than 6 mm, condition which is respected taking into account the dimensions previously presented. The height of the honeycomb is also 80 mm, making the structure a cubic specimen.

**Figure 8:**
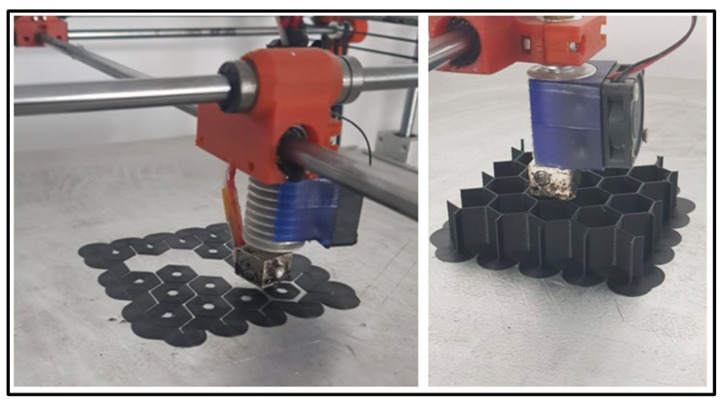
Honeycomb compression specimen realized by FDM manufacturing.

The 3D printed test specimens have been realized from the same CPE elastomer with embedded milled carbon microfibers, representing basically a section of the core of the tail rotor blade. The thermoplastic extrusion manufacturing process is presented in detail in Figure 8. The bottom material layer was placed in order to facilitate component removal without any deteriorations.

**Figure 9:**
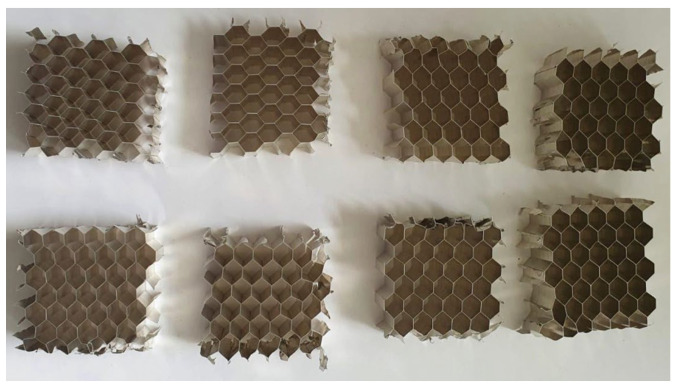
Compression test specimens made from 5052 aluminum alloy.

The cubic specimens made from 5052 aluminum alloy were extracted from a larger NIDA honeycomb panel, as to respect the general dimension requirements of the testing standard. The metal honeycomb cell dimensions match those presented in Figure 1. The comparability of the two types of material was realized by ensuring an identical compression contact surface for both aluminum and CPE specimens. The eight aluminum alloy test specimens subjected to compression testing can be visualized in Figure 9.

**Figure 10:**
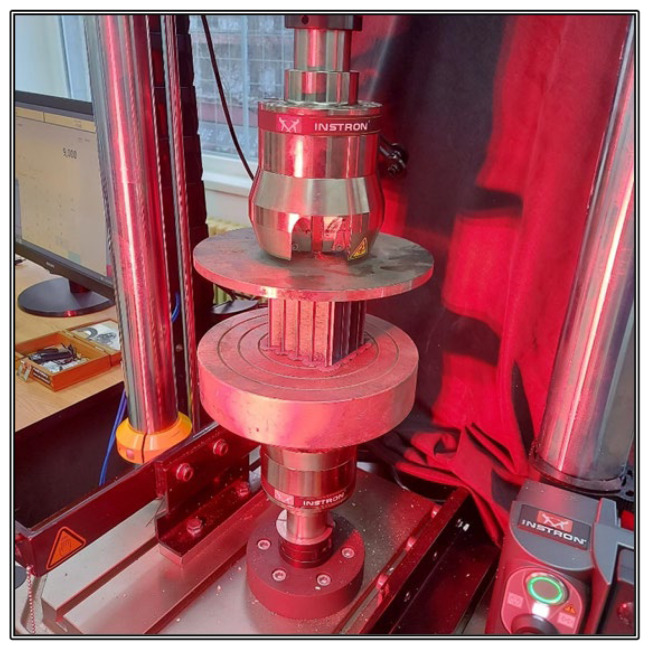
Compression test setup.

The experimental set-up consisted of the Instron 8872 axial testing system, equipped with two horizontal plates at the specimens’ upper and lower extremities. They were positioned parallel to each other and perpendicular to the sides of the specimens in such a manner that the load is uniformly distributed across the specimen contact surface (Figure 10). The main compressive characteristic determined from the experimental test is the compressive modulus, derived from the stress-strain evolution.

The performance of the honeycomb specimens was evaluated with the Digital Image Correlation system, with the specimens being places between the plates of the Instron 8872 testing equipment, produced by Instron, in Norwood, MA, USA. The compressive strength of the specimens can be determined by knowing the ultimate load (*P*) and the cross-sectional area (*A*) of the specimen:

(6) $$\sigma \;=\;\frac{P}{A}$$

The flatwise compressive modulus can be calculated from the experimental results, with the following expression:

(7) $$E\;=\;S\cdot \frac{t}{A}$$

where “*S*” is the slope of the initial linear portion of the load-deflection curve and “*t*” is the core thickness.

## 3. Results Presentation and Interpretation

The specimens have been subjected to tensile and compression testing in order to obtain a comprehensive description of the mechanical characteristics of the honeycomb material employed for the future composite tail rotor blade. The results have been presented for each specimen type, by underlining the main advantages and disadvantages of the material and with reference to the manufacturing direction.

### 3.1. Assesing the Ortothropic Nature of the Chlorinated Polyethylene

#### 3.1.1. Tensile Specimen Evaluation—XZ1 Specimens

The first four tensile tests specimens have been constructed on the surface of the machine’s printing pad, as displayed in Figure 4a. This printing orientation allows for a better contour definition, resulting in more qualitative specimens.

**Figure 11:**
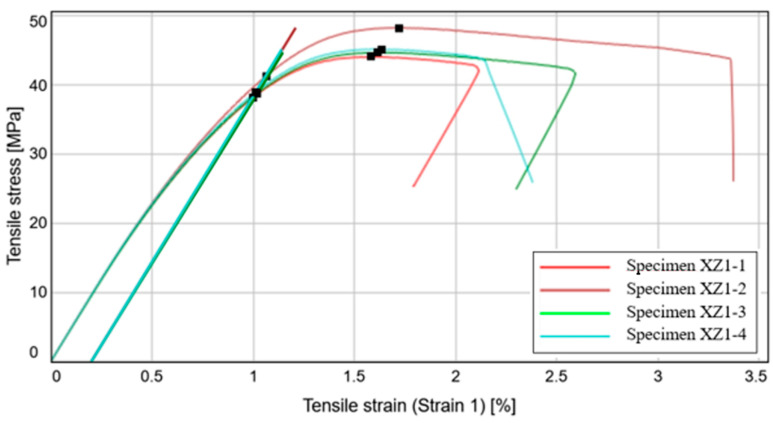
Stress-Strain curve variation for the XZ1 specimens.

In Figure 11 we can see the stress-strain curves of the first four tensile test specimens which are characterized by a very similar behavior on the 0.05–0.15% segment of the slope, as a result of a low-density variation in the samples and a relative constant modulus of elasticity of approximately 4783.22 MPa. Compared to the two directions of printing which will be presented later on, it is important to state that this direction displays the highest modulus of elasticity.

The 0.2% offset yield strength, presented in the third column of Table 3, displays relatively high values at which the 0.2% plastic deformation occurs. The importance of this parameter resides in the fact that it is frequently quoted by material suppliers and also, in the engineering domain, as a measure of the material resistance. The median tensile stress has a value of 44.97 MPa, with a very small standard deviation of only 1.87 MPa, the failure occurring at roughly the same tensile strain for all of the four specimens.

The coefficient of variation for all of the parameters presented in Table 3 is situated below 5%, indicating an accurate set of experimental results, with very small variations. This in turn, resides in the fact that the fused deposition modeling process is qualitative, producing specimens with very similar characteristics.

**Figure 12:**
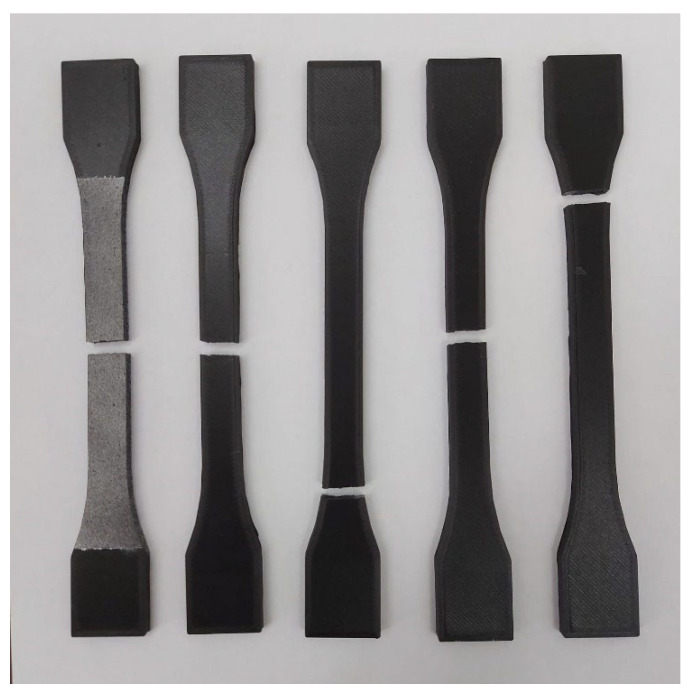
Failure mode of the XZ1 specimens.

By comparing the results with CPE CF112 Carbon datasheet it can be stated that the modulus of elasticity is more than two times larger than the one stated by the filament manufacturer, for the current printing direction of the specimen. On the other hand, the median yield strength is 34% lower than the stated value. Taking into account the fact that this manufacturing direction has the highest modulus of elasticity, it can be deduced that the materials constructed in this direction will better retain their initial geometrical properties under the effect of tensile loads. The failure mode of the five specimens is predominantly central, as can be seen in Figure 12.

#### 3.1.2. Tensile Specimen Evaluation—XY Specimens

The second manufacturing direction is on the lateral longitudinal side of the specimens, corresponding to the posture presented in Figure 4b. This setting differs from the other two printing directions, on the grounds that it makes use of printing supports to help preserve the geometrical characteristics of the specimens, which will be removed after the process is finished.

**Figure 13:**
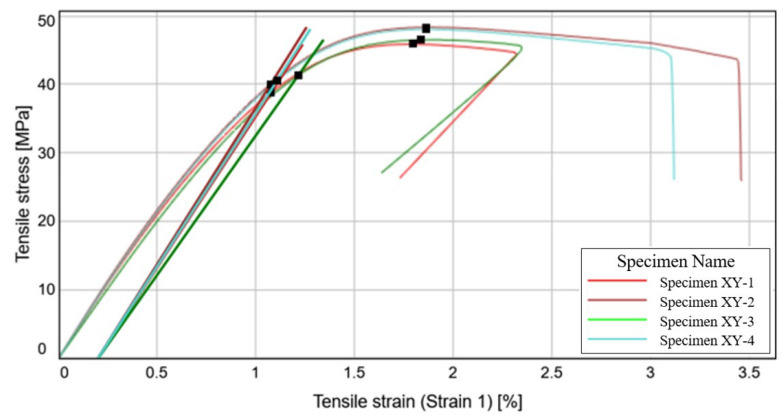
Stress-Strain curve variation for the XY specimens.

From the stress-strain curves depicted in Figure 13, we can observe that the second and third specimens demonstrate a high resilience, due to the fact that material failure occurs above a tensile strain of 3%. This durability is most likely a result of the milled carbon fibers reinforcing the filament, which have a stronger effect on this direction of manufacturing.

With an average elongation of roughly 85% more than the initial specimen length, the results indicate a high degree of elasticity, which is an essential characteristic for aircraft applications. The highest average tensile stress has been measured for this printing direction, according to the data presented in Table 4. With an average tensile stress of 47.29 MPa, it is 50% more resistant that the vertical printed specimens and approximately 5.15% more resistant than the specimens printed on the flat surface. The tensile strain at the moment of failure is roughly the same for all the four specimens.

With relatively small standard deviations and coefficients of variation, the obtained results are within an acceptable limit to be considered as precise and reliable. In relation to the CPE CF112 Carbon datasheet, the experimental results are similar to those obtained for the previously studied specimen, manufactured on the printed pad. It is safe to say that these two manufacturing directions provides the specimen with a relatively similar mechanical behavior.

**Figure 14:**
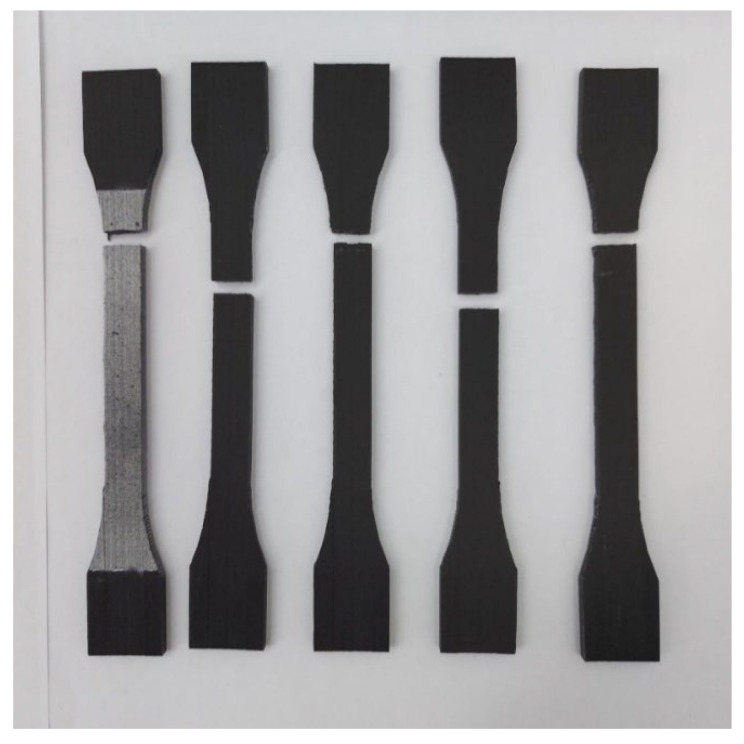
Failure mode of the XY specimens.

On account of the largest identified median tensile stress, it can be highlighted that the materials printed in this direction have a higher endurance to tensile loads than the other manufacturing directions. The broken specimens are displayed in Figure 14, after the tensile testing process.

#### 3.1.3. Tensile Specimen Evaluation—XZ2 Specimens

The third and final type of tensile specimen was realized in a vertical straight position, as is presented in Figure 4c. Taking into account the manufacturing process, the overall strength of the specimen represents in fact the strength of the adhesion between the overlapping layers of material. This posture offers a slightly better bond than the other two, due to the fact that the weight of the upper layers pushes down on the lower layers, thus compressing the material and eliminating potential interior voids.

After a certain threshold of about 29.5 MPa is surpassed, debonding occurs and the specimen ruptures in the area where the shear stress reaches a maximum value. In the light of a rigorous manufacturing process, this phenomenon takes place when the tensile force breaks the material bond, but otherwise, fabrication errors, such as inadequate curing time, temperature variation or porosity apparition could be the triggering factor.

**Figure 15:**
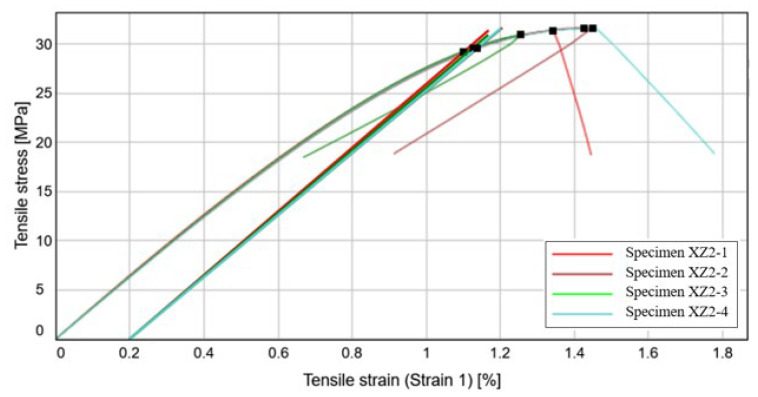
Stress-Strain curve variation for the XZ2 specimens.

Figure 15 depicts the variation of the tensile stress with the tensile strain of the four tested specimens. The yield strength corresponding to a 0.2% plastic strain has a mean value of approximately 29.56 MPa, the normal stress is around 31.50 MPa, whilst the tensile strain exhibits an elongation ranging from a quarter of the initial specimen length, up till a maximum of 45%, as presented in Table 5. Compared to the previous printing directions, the vertical direction is characterized by a quick failure, after the ultimate tensile strength is reached.

In terms of the tensile modulus, the specimen printed in this direction has an elasticity modulus 29.62% smaller than the specimens printed on the lateral longitudinal side and by 33.62% smaller than the specimens manufactured on the printing pad.

The variation coefficient has a value below 1.40% for the first three properties displayed in Table 5, whilst the tensile strain has a maximum variation of 6.53%. The values obtained are relatively low which would indicate that the methods and results are of a relatively high precision and trustworthy. In comparison with CPE CF112 Carbon datasheet it can be stated that although the modulus of elasticity has a closer value to that indicated by the producer, the general performances are somewhat lower, especially due to the filament disposition.

**Figure 16:**
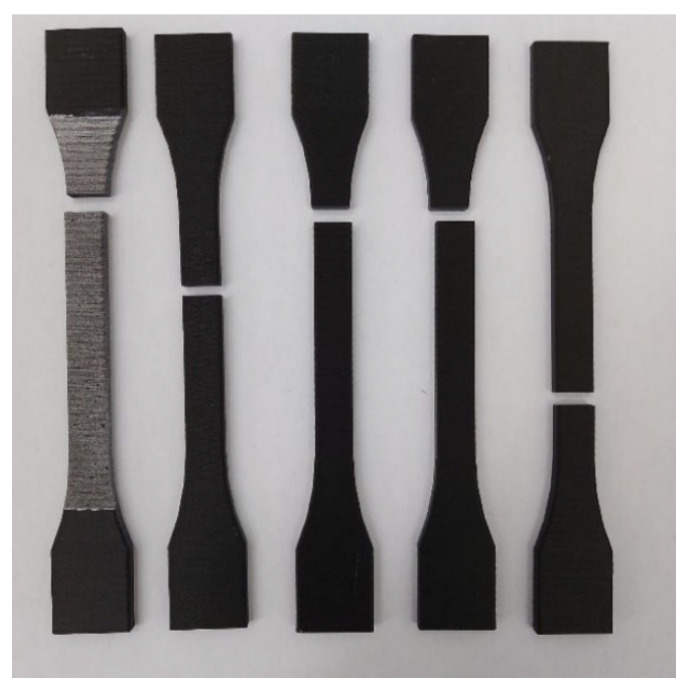
Failure mode of the XZ2 specimens.

By comparison with the other two specimen types, the mechanical properties are significantly lower and if possible, it would be advisable to avoid the positioning this manufacturing direction in the pathway of the applied tensile loads. The failure mode is presented in Figure 16.

#### 3.1.4. Tensile Properties Evaluation Using the DIC System

**Figure 17:**
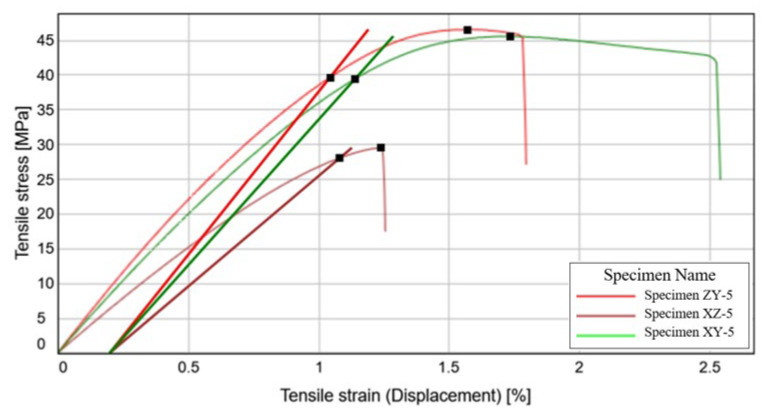
Stress-Strain curve variation for each 3D printed specimen type.

The three previous experimental data sets where supplemented with the specimens evaluated through the Digital Image Correlation method. For this purpose, a specimen for each of the three-manufacturing directions has been employed. The stress-strain curves of these tensile tests are presented in Figure 17.

The purpose of this alternative measuring technique is to see if there is any notable difference in the way the two methods determine de mechanical properties of the material and also to increase the reliability of the test methods utilized and of the results obtained.

The values obtained from the Instron hydraulic testing machinery equipped with the extensometer have been compared with those from the Digital Image Correlation system, as displayed in Table 6. By comparing the two data sets, it can be underlined that the tensile strain measured with the longitudinal extensometer is larger than that measured with the Digital Image Correlation method. In terms of other mechanical characteristics, it can be stated that there is no general tendency of higher or lower measurements for neither method.

The values obtained using the Digital Image Correlation method are of comparable value with the measurements determined with the extensometer, with the general remark that the tensile modulus values are positioned in the minimum-maximum previously determined interval, while the 0.2% yield strength and the tensile strength have slightly lower values when using the Q400 DIC system.

The fifth specimen of each printing directions confirms the previously underlined conclusions for each individual experimental case. The manufacturing direction of the XZ1 tensile specimens exhibits the best performances in terms of tensile resistance and will constitute the basis for manufacturing the honeycomb structure. Poisson’s ratio for this direction was determined at 0.316, which is somewhat of a medium value between the other two manufacturing directions. Altogether, the data collected using the Digital Image Correlation system are in close relation with those determined using the extensometer, and will be taken into consideration when assessing the overall mechanical characteristics of the studied materials.

### 3.2. Compression Properties Evaluation of the Chlorinated Polyethylene Honeycomb

**Figure 18:**
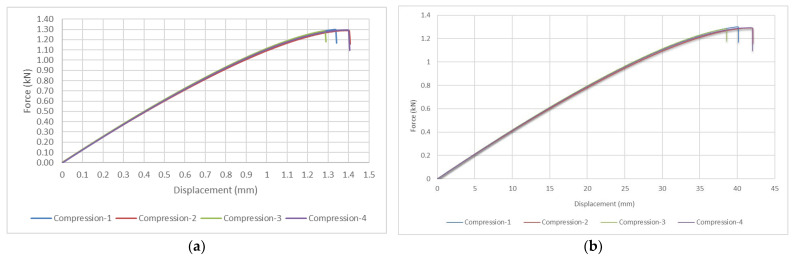
CPE honeycomb compression tests: (**a**) Force-displacement graphical evolution (**b**) Force-time graphical evolution.

The four compression test specimens where subjected to the same uniformly applied force. Figure 18 presents on one side, the variation of the force applied by the load cell with the vertical displacement and on the other side, the force-time curves. The graphic presents a similar linear evolution for all the tested specimens, with an average maximum compression force of 1.3614 kN and a mean maximum displacement of approximately 1.35 mm. Taking into account that the initial height of the honeycomb samples was approximately 80 mm, we can deduce that the vertical load produced a compression of 1.65% of the total dimension. The displacement speed of the load cell was 2 mm/min, respectively 0.0325 mm/s. The failure condition of the specimens was reached after an average duration of approximately 38 s.

The specimens display a similar mechanical behavior in all four tryouts. The minimum stress was identified for the third specimen, with a value of 1.2567 MPa, while the maximum stress was measured for the fourth specimen at a value of 1.4617 MPa. In practical applications, the real values for the stress and strains are more adequate to be utilized, rather than the engineering values. Thus, the real stress can be determined by dividing the compression force to the instantaneous cross-sectional area of material (A), as opposed to the original cross-sectional area ($A_{0}$), which is used for the engineering stress. The mathematical expression is:

(8) $$\tilde{\sigma }\;=\;\frac{F}{A}\;=\;\sigma \frac{A_{0}}{A}$$

Corresponding to the real stress, there is also a real value for the strain, which can be determined with the following formula:

(9) $$\varepsilon^{˜}\;=\;ln\left(1+\varepsilon \right)$$

**Figure 19:**
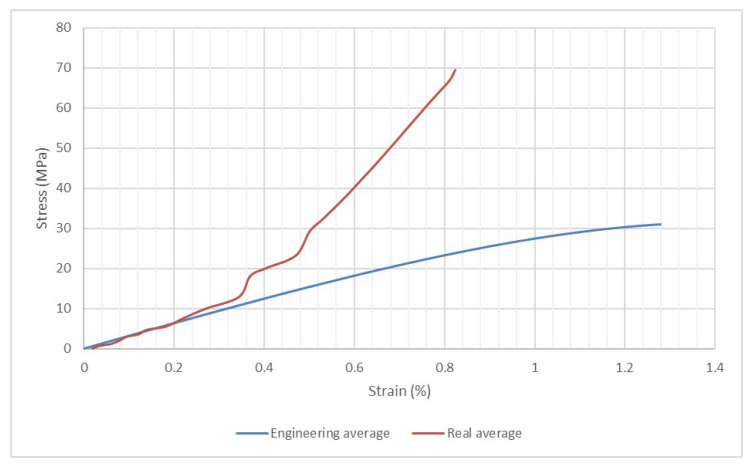
Comparison between the stress-strain evolution for the real values and the engineering values.

By making use of the previous mathematical expressions, a comparison between the stress-strain curves for the real and the engineering values has been depicted in Figure 19, as mean values of the four compression tests.

The previous figure presents a similar evolution of the stress-strain curves, below a strain value of 0.2%. The maximum real stress value is approximately 70 MPa, which is double the value of the maximum engineering stress. The difference is most likely a result of the buckling effect on the walls of the honeycomb specimen.

**Figure 20:**
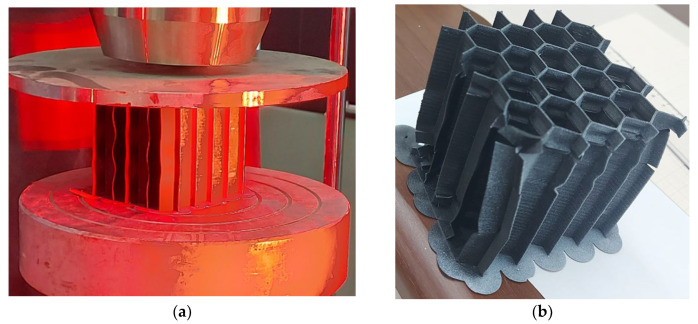
Compression test of CPE honeycomb specimen (**a**) Buckling of external walls (**b**) Specimen after failure.

In the compression testing process, the first visual effect revealed is the buckling of the external walls of the honeycomb, which are not a part of any hexagonal cell. The buckling effect and the failure mode of the honeycomb specimens can be observed in Figure 20.

Material failure is characterized by debonding alongside the vertical length of the hexagonal cells. This effect is especially visible in the outer walls of the honeycomb, where the adhesion weakened between the extruded layers of material and the deformation reached a maximum value. The rigidity of the material is sufficiently high so that the layers of material are not crushed under the compression force.

By knowing that the area of the compressed surface is 433.34 mm^2^, from the finite element model used for 3D printing, the compressive modulus and the compression stress can be determined by using the formulas 5 and 6 from the ASTM C365 standard. The cross-sectional area was determined by measuring the thickness and the length of the walls of the honeycomb, multiplied by the number of walls.

The material deformation had a progressive manifestation, being directly proportional with the compressive force across the whole length of the experimental tryout. The main mechanical characteristics determined with the Digital Image Correlation system are presented in Table 7.

The experimental results produced a compressive modulus of nearly 3200 MPa, which is higher than the value of 2200 MPa stated by the filament manufacturer in the material data sheet. This result is a clear indicator that the material has a better performance handling compression loads as opposed to tensile loads. The material does not exhibit a brittle behavior, being suitable for employment as a honeycomb core.

The dispersed milled carbon fibers in the filament contribute to the overall tensile resistance of the material. The ultimate compression stress is approximately 31.40 MPa, relatively close to the ultimate tensile stress presented in the data sheet.

### 3.3. Mechanical Properties Comparison between the CPE Honeycomb and the 5052 Aluminum Alloy Honeycomb

Having determined the mechanical properties of the previous material which is proposed for manufacturing the honeycomb core of a composite tail rotor blade, it is important to know how the 5052 aluminum allow handles compression loading, in order to have a better view of the advantages and disadvantages associated with the employing the CPE CF112 Carbon material.

**Figure 21:**
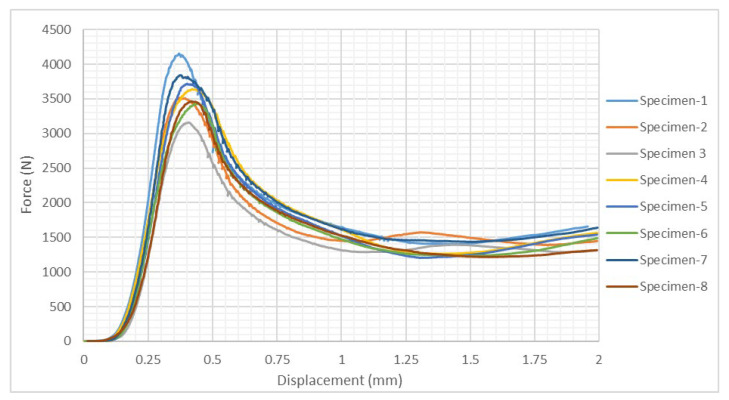
Force-displacement curves for the compression testing of the 5052 aluminum alloy honeycomb specimens.

The compression testing of the 5052 aluminum alloy honeycomb was realized in the same conditions as the CPE honeycomb. A total of eight honeycomb specimens were extracted from a larger NIDA 2.3-3/8-.0015 P(5052) panel. The specimens where carrefully dimensioned in order to have knowledge of the compression contact surface for both CPE and aluminum alloy honeycombs. The force-displacement curves for each of the eight specimens are visibile in Figure 21.

As the previous graphic exhibits, the tests resulted in similar force-strain curves for all eight study cases, with sligth variations in the intensity of the compression force. The compression force peaked at an average strain of 0.401%, precisely 12.22 s after the tryout commenced. Beyond this maximum threshold the material displays a plastic behaviour, characterized by a progressive decrease of the force under an increasing strain, untill in stabilized around the value of 1.5 kN. The compression modulus and the ultimate stress of each specimen has been determined by using expressions 5 and 6 and are presented in Table 8, along with other measured parameters.

Taking into account that the variation coefficient is below 9% for the maximum compression force, we can conclude that the NIDA aluminum alloy specimens exhibit a constant mechanical behaviour and the results have a high degree of precision.

Knowing the areas for the two types of honeycomb specimes, a comparison can be made between their individual performances. Thus, having measured the compression properties for each material type, an evaluation of the mechanical properties can be realized based on the values presented in Table 9. The main experimental characteristics are presented as average values for each individual material.

Comparing the mechanical properties previously presented, the following conclusions can be underligned referred to the studied materials:

- the 5052 aluminum alloy is characterized by a higher compressive strength due to the higher ultimate compression stress reached in the linear elastic region of the material;
- the metal honeycomb is more rapidly placed under tension, reaching its maximum compression limit at less that a third than the CPE honeycomb, consisting of a quicker material failure;
- the CPE honeycomb is more resilient than the aluminum alloy, in the sense that it can material failure is reached at a higher mechanical strain;
- the CPE material is much lighter than the aluminum alloy, a fact which results in a significant mass reduction; the CPE honeycomb’s mass represents only 34.1% that of the aluminum alloy specimen, an ideal advantage when manufacturing aircraft components.

Taking into account the previous remarks, it is safe to say that the chlorynated polyetilene reinforced with carbon microfibers is a satisfactory alternative for the 5052 aluminum alloy, due to it’s mechanical properties which makes it suitable for aircraft components such as honeycomb cores. The overall mechanical properties of the CPE CF112 Carbon which have resulted from the previously presented test sets, are depicted in Table 10.

### 3.4. Finite Element Analysis – Results Comparison: Experimental vs. FEA

Finite element simulations were performed in order to reproduce the compression loading of the two different materials, by using static structural analyses carried out in Ansys Workbench version 19.2, as the main analysis software. The geometrical models where realized with a solid element structure in Catia V5, are where meshed with tetrahedral elements. The step controls setting of the analyses are defined as to facilitate solution convergence, with the following values: 50 initial substeps, 25 minimum substeps and 200 maximum substeps. The two numerical analyses were performed with the same boundary conditions, ensuring a precise correlation between the physical model and the finite element model. The specimens were locked with a remote displacement on the lower surface of the model and the mean vertical displacement was applied on the upper face of the model.

The 5052 aluminum alloy and the CPE CF112 Carbon were defined in the finite element analysis software by using the following experimentally determined properties:

- tensile modulus;
- compression modulus;
- Poisson’s ratio;
- real values for stress and strain, defined as a set of uniaxial test data.

**Figure 22:**
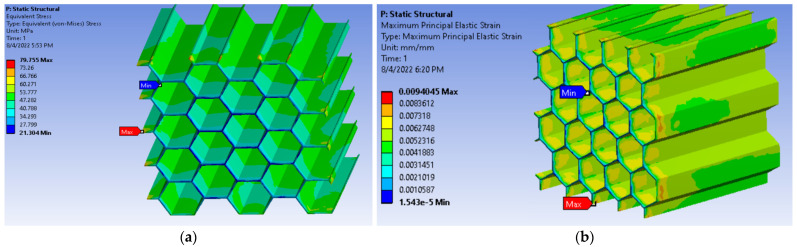
Results determined for the FEA: (**a**) Equivalent stress (von-Mises stress) for the CPE CF112 Carbon; (**b**) Equivalent strain for the CPE CF112 Carbon.

Following the application of the vertical displacement for both models, the equivalent von-Mises stress and the equivalent strain were determined, their distribution on the geometrical models being presented in Figure 22.

The color code of the stress distribution clearly highlights the deflected shape of the honeycomb walls, corresponding to a wavelike pattern. The figures depict the fact that the stress and strain maximum values are concentrated in the lower side of the honeycomb. The validation methodology implied determining the reaction force of the model after applying the maximum displacement, which will be compared with the experimental force measured by the load-cell. The comparison between the FEA results and the real values of the experimental tests is realized in Table 11.

The finite element analysis results offer a satisfying similarity with the experimentally obtained values, which were transformed into real values by using expressions 7 and 8. The resulting reaction force is approximately 12% lower than the experimental value, suggesting that the material definition and the 3D model is sufficiently accurate in order to be inserted as a honeycomb core in the model of the desired tail rotor blade.

## 4. Conclusions

The experimental study was carried out in order to have a complete understanding of the CPE material performances and its capability to be implemented as a honeycomb core material. The study has brought about satisfying results in terms of accuracy, from the standard deviation and coefficient of variation point of view.

The honeycomb realized through the fused deposition modeling process appears to be efficient enough to withstand the action of compressive loads, having in mind the fact that the spar and the skin of the composite tail rotor blade will be manufactured from epoxy resin reinforced with high resistance carbon fiber. The capability of the fused deposition modeling process to realize complex geometries, such as hexagonal cells, and the significant weight reduction it implies compared with traditional laminar composite materials, are key advantages which make the final product satisfactory.

The optimal manufacturing direction has been identified after determining the tensile properties of the thermoplastic polymer in all three main printing directions, which will be used in constructing both the honeycomb specimens, as well as the honeycomb core of the composite tail rotor blade. The obtained results were of similar value with the data stated by the producer in the material datasheet.

Although the results comparison between the 5052 aluminum alloy honeycomb and the CPE CF112 Carbon honeycomb suggest that the metal core is more resistant, the CPE core compensates through other important advantages, such as: mass reduction, corrosion and chemical resistance, rapid manufacturing, etc.

The compression tryouts were reproduced in a finite element analysis and were validated using the experimentally determined results. The results display a relative high degree of precision and thus, will serve as a basis for manufacturing the blade and testing the overall structure, both experimentally and also by computational analysis.

## Figures and Tables

**Table 1:** Main mechanical properties of the honeycomb materials.

Aluminum Alloy 5052 [35]	CPE with Milled Carbon Fibres [36]
Density—2680 kg/m^3^	Reference—CPE CF112 Carbon
Tensile strength— 228 MPa	Polymer base—co-polyester
Yield strength— 193 MPa	Manufacturer—Filamentum Manufacturing
Tensile modulus— 70.3 GPa	Resistance against acids, alkalis, alcohols (at 25 °C)—good
Poisson’s ratio— 0.33	Resistance against water, acetone, oils, greases, car fluids, ozone (at 25 °C)— medium to low
Shear strength— 138 MPa	Print temperature (°C)— 250–270
Shear modulus— 25.9 GPa	Rotor blade application—Honeycomb
Melting temperature— 607—649 °C	Tensile strength at Yield—52.4 MPa
Specific heat— 0.88 J/g-°C	Tensile strength at Break—37.7 MPa
Thermal conductivity— 138 W/m-K	Material density— 1.16 g/cm^3^
	Tensile Modulus— 2200 MPa

**Table 2:** Manufacturing characteristics of the Custom 3D Xcub 3D printer.

Optimum printing speed	50 mm/s
Optimum traveling speed	50 mm/s
Extruder type	0.4 mm dual extruder
Heated printing pad	0.6 kW power with a maximum heating temperature of 200 °C
Structure	Aluminum Bosch Rexroth profile
Maximum printing volume	42 × 42 × 47 cm
Maximum power consumption	0.7 kW

**Table 3:** Mechanical characteristics obtained for the XZ1 specimens.

Specimen Label	Modulus (Segment 0.05–0.15%) [MPa]	Tensile Stress at Yield (Offset 0.2%) [MPa]	Ultimate Tensile Strength [MPa]	Tensile Strain (Strain 1) at Tensile Strength [%]
Specimen XZ1-1	4782.89	38.11	44.10	1.58
Specimen XZ1-2	4783.56	41.26	48.31	1.72
Specimen XZ1-3	4729.04	38.76	44.73	1.61
Specimen XZ1-4	4801.04	38.95	45.22	1.64
Median value	4783.22	38.86	44.97	1.62
Standard deviation	31.22	1.38	1.87	0.06
Coefficient of variation	0.65	3.51	4.11	3.65

**Table 4:** Mechanical characteristics obtained for the XY specimens.

Specimen Label	Modulus (Segment 0.05–0.15%) [MPa]	Tensile Stress at Yield (Offset 0.2%) [MPa]	Ultimate Tensile Strength [MPa]	Tensile Strain (Strain 1) at Break [%]
Specimen XY-1	4426.07	38.76	45.86	1.80
Specimen XY-2	4576.66	40.02	48.36	1.86
Specimen XY-3	4078.94	41.29	46.52	1.84
Specimen XY-4	4470.12	40.50	48.06	1.86
Median value	4448.10	40.26	47.29	1.85
Standard deviation	215.49	1.06	1.20	0.03
Coefficient of variation	4.91	2.65	2.54	1.66

**Table 5:** Mechanical characteristics obtained for the XZ2 specimens.

Specimen label	Modulus (Segment 0.05–0.15%) [MPa]	Tensile Stress at Yield (Offset 0.2%) [MPa]	Ultimate Tensile Strength [MPa]	Tensile Strain (Strain 1) at Break [%]
Specimen XZ2-1	3239.24	29.18	31.39	1.34
Specimen XZ2-2	3148.39	29.54	31.65	1.43
Specimen XZ2-3	3196.41	29.60	30.94	1.25
Specimen XZ2-4	3155.54	29.59	31.60	1.45
Median value	3175.97	29.56	31.50	1.38
Standard deviation	41.95	0.20	0.33	0.09
Coefficient of variation	1.32	0.69	1.04	6.53

**Table 6:** Comparison between values obtained with DIC. measurement system and digital extensometer.

Specimen Label	Modulus (Segment 0.05–0.15%) [MPa]		Tensile Stress at Yield (Offset 0.2%) [MPa]		Ultimate Tensile Strength [MPa]		Tensile Strain (Strain 1) at Break [%]	
	DIC Value	Extensometer Mean Value	DIC Value	Extensometer Mean Value	DIC Value	Extensometer Mean Value	DSIC Value	Extensometer Mean Value
Specimen XZ1-5	4703.16	4783.22	39.70	38.86	46.60	44.97	1.57	1.62
Specimen XZ2-5	3186.80	3175.97	28.08	29.56	29.56	31.50	1.24	1.38
Specimen XY-5	4199.19	4448.10	39.44	40.26	45.62	47.29	1.73	1.85

**Table 7:** Mechanical characteristics of honeycomb CPE subjected to compressive testing.

Specimen label	Maximum Force [kN]	Maximum Vertical Displacement [mm]	Compression Modulus [Pa]	Ultimate Compression Stress [MPa]	Strain [%]
Compression-1	1.3017	1.3349	3239.24	31.39	1.37
Compression-2	1.2921	1.3862	3148.39	31.65	1.43
Compression-3	1.2858	1.2796	3196.41	30.94	1.25
Compression-4	1.2957	1.3949	3155.54	31.60	1.45
*Average values*	*1.293825*	*1.3489*	*3184.90*	*31.40*	*1.38*

**Table 8:** Mechanical characteristics of 5052 aluminum alloy honeycomb subjected to compression testing.

Specimen Label	Maximum Force [N]	Maximum Vertical Displacement [mm]	Compression Modulus [MPa]	Ultimate Compression Stress [MPa]	Strain [%]
Specimen-1	4154.64	0.3699	24,790.44	95.46	0.3694
Specimen-2	3515.08	0.372	21,170.95	80.76	0.3711
Specimen-3	3160.62	0.4073	16,606.61	72.62	0.4068
Specimen-4	3639.29	0.4213	20,534.92	83.62	0.4212
Specimen-5	3718.23	0.4022	19,621.32	85.43	0.4023
Specimen-6	3435.15	0.4431	18,261.02	78.93	0.4423
Specimen-7	3841.54	0.3747	21,963.23	88.27	0.3750
Specimen-8	3466.36	0.4184	17,363.97	79.64	0.4190
*Average values*	*3617.41*	*0.3926*	*20,039.06*	*83.12*	*0.3921*

**Table 9:** Comparison between the average performances of the two honeycomb materials.

Specimen Type	Average Maximum Compression Force (kN)	Maximum Displacement at Maximum Force (mm)	Time at Maximum Compression Force (s)	Mean Strain at Maximum Compression Force (%)	Mean Ultimate Compression Stress (MPa)
CPE CF112 Carbon honeycomb	1.294	1.3489	40.45	1.380	31.40
5052 aluminum alloy honeycomb	3.616	0.3926	12.22	0.3921	83.12

**Table 10:** Experimentally determined mechanical properties for the CPE CF112 Carbon material.

Test Type	Specimen Type	Mechanical Property
*Tensile test*	XY Specimens	Tensile modulus—4448.10 MPa
		Tensile stress at yield (0.2%)—40.26 MPa
		Ultimate tensile stress—47.29 MPa
		Tensile strain at tensile strength—1.85%
		Poisson’s ratio—0.388
*Tensile test*	XZ1 Specimens	Tensile modulus—4783.22 MPa
		Tensile stress at yield (0.2%)—38.86 MPa
		Tensile stress at tensile strength—44.97 MPa
		Tensile strain at tensile strength—1.64%
		Poisson’s ratio—0.316
*Tensile test*	XZ2 Specimens	Tensile modulus—3175.97 MPa
		Tensile stress at yield (0.2%)—29.56 MPa
		Tensile stress at tensile strength—31.50 MPa
		Tensile strain at tensile strength—1.38
		Poisson’s ratio—0.225
*Compression test*	-	Flatwise compressive modulus—3184.90 MPa
		Compression stress at yield (0.2%)—29.48 MPa
		Ultimate compression strain—31.40 MPa
		Strain at ultimate compression strain—1.38%

**Table 11:** Comparison between the FEA and experimental results for the CPE CF112 honeycomb.

Evaluated Property	Experimental Results	FEA Results	Error
Maximum displacement (mm)	1.3489	1.3489	-
Maximum compressive force (kN)	1.293	1.438	11.21%
Equivalent stress (MPa)	79.755	70.719	12.77%
Equivalent strain (%)	0.823	0.940	14.21%

## Data Availability

Not applicable.
